# Hydatidose de la loge des adducteurs de la cuisse: un cas rare dans la littérature

**DOI:** 10.11604/pamj.2016.24.76.9571

**Published:** 2016-05-24

**Authors:** Adil El Alaoui, Amine mezzani

**Affiliations:** 1 Service de Chirurgie Orthopédique (A) du Centre Hospitalier Universitaire Hassan II de Fès, Maroc

**Keywords:** Hydatidose, loge des adducteurs, cuisse, Hydatid disease, adductors lodge, thigh

## Image en médecine

La localisation de prédilection de l'échinococcose humaine est le foie et les poumons dont elles représentent 85% des cas. L'atteinte des parties molles est exceptionnelle. Nous rapportons un cas rare d'un kyste hydatique de la loge des adducteurs de la cuisse chez un patient de 42 ans, d'origine rurale avec notion de contact prolongé avec les chiens qui présente depuis 6 mois des douleurs inguinales avec des tuméfactions au niveau de la face interne de la cuisse droite augmentant progressivement de volume, le tout évoluant dans un contexte d'apyrexie et de conservation de son état général. Après 3 mois d'évolution ; la douleur est devenue inflammatoire et insomniante avec apparition des fistules en regard des masses ramenant des vésicules blanchâtres (A, B). Le bilan biologique a révélé une hyperéosinophilie. Le patient a bénéficié d'une IRM qui a objectivé la présence de nombreux kystes hydatiquesau niveau de loge des adducteurs de la cuisse droite (C, D). une biopsie exérèse des kystes et des membranes restantes a été réalisée (E, F) dont les résultats anatomo-pathologiques sont revenus en faveur d'une hydatidose de la cuisse. Le traitement chirurgical a été enchainé par un traitement médical à base d'Albendazol pendant 28 jours avec une bonne évolution clinique et radiologique.

**Figure 1:**
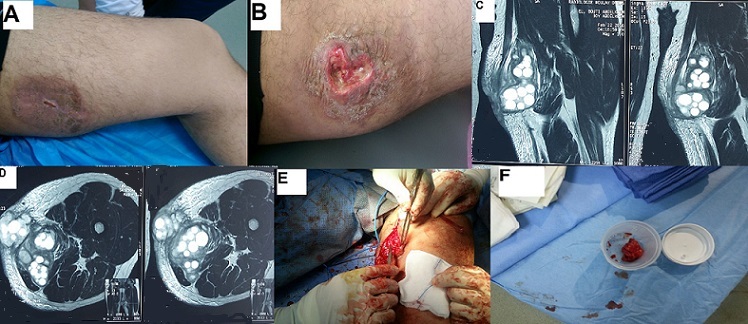
A) fistule d'un kyste hydatique au niveau de loge des adducteurs de la cuisse; B) issu de membranes blanchâtres en regard de la fistule; C) coupe sagittale sur IRM de la cuisse montrant une hydatidose au niveau de loge des adducteurs; D) coupe coronale sur IRM de la cuisse montrant une hydatidose au niveau de loge des adducteurs; E) exérèse des kystes hydatiques rompus; F) aspect macroscopique des membranes des kystes hydatique

